# QALY losses for chronic diseases and its social distribution in the general population: results from the Belgian Health Interview Survey

**DOI:** 10.1186/s12889-022-13675-y

**Published:** 2022-07-07

**Authors:** Lisa Van Wilder, Brecht Devleesschauwer, Els Clays, Johan Van der Heyden, Rana Charafeddine, Aline Scohy, Delphine De Smedt

**Affiliations:** 1 Department of Public Health and Primary Care, Ghent University, University Hospital, Ghent, Belgium; 2 Department of Epidemiology and public health, Sciensano, Brussels, Belgium; 3 Department of Translational Physiology, Infectiology and Public Health, Ghent University, Merelbeke, Belgium

**Keywords:** Quality-adjusted life year, Health-related quality of life, Health inequality, Chronic disease, EQ-5D

## Abstract

**Background:**

The burden of chronic diseases is rapidly rising, both in terms of morbidity and mortality. This burden is disproportionally carried by socially disadvantaged population subgroups. Quality-adjusted life years (QALYs) measure the impact of disease on mortality and morbidity into a single index. This study aims to estimate the burden of chronic diseases in terms of QALY losses and to model its social distribution for the general population.

**Methods:**

The Belgian Health Interview Survey 2013 and 2018 provided data on self-reported chronic conditions for a nationally representative sample. The annual QALY loss per 100,000 individuals was calculated for each condition, incorporating disease prevalence and health-related quality of life (HRQoL) data (EQ-5D-5L). Socioeconomic inequalities, based on respondents’ socioeconomic status (SES), were assessed by estimating population attributable fractions (PAF).

**Results:**

For both years, the largest QALY losses were observed in dorsopathies, arthropathies, hypertension/high cholesterol, and genitourinary problems. QALY losses were larger in women and in older individuals. Individuals with high SES had consistently lower QALY loss when facing a chronic disease compared to those with low SES. In both years, a higher PAF was found in individuals with hip fracture and stroke. In 2013, the health inequality gap amounts to 33,731 QALYs and further expanded to 42,273 QALYs in 2018.

**Conclusion:**

Given that chronic diseases will rise in the next decades, addressing its burden is necessary, particularly among the most vulnerable (i.e. older persons, women, low SES). Interventions in these target groups should get priority in order to reduce the burden of chronic diseases.

**Supplementary Information:**

The online version contains supplementary material available at 10.1186/s12889-022-13675-y.

## Background

Chronic diseases remain among the greatest public health concerns worldwide as the prevalence continues to increase due to population aging [1, 2]. As a result, the burden of chronic diseases is rapidly rising, both in terms of morbidity and mortality. With an estimated 41 million deaths annually, chronic diseases are responsible for 73% of total global deaths [2]. Nowadays, public health policies demand knowledge on all aspects of health because mortality measures alone are insufficient to fully capture disease burden [3]. Indeed, chronic diseases impose a high morbidity burden for patients, caregivers, and the entire society [4, 5]. Consequently, health-related quality of life (HRQoL) has gained importance over the past decades as it captures patients’ self-perceived physical, mental, and social impact of a medical condition, its symptoms and treatment [5, 6]. Ideally, health care policies target both quantity and quality of life.

Several measures exist for measuring burden of chronic diseases that reflect both quantity and quality of life, such as the frequently used quality-adjusted life year (QALY). QALYs measure the impact of disease on mortality and morbidity into a single index, allowing to assess the burden of individual diseases at population level [1, 7, 8]. Moreover, QALYs can simplify the complexity of chronic diseases and enable direct comparisons of the relative impact of diseases [8]. QALY loss in chronic diseases has already been explored in several studies; however, estimates for specific diseases with low prevalence often not detectable in smaller studies, are scarce [1, 8–10]. Furthermore, it is well-known that low socioeconomic status (SES) is associated with higher disease costs (due to poorer insurance schemes) and poor health outcomes [11]. Searching for socioeconomic inequalities in QALY losses is therefore important to support policy guidelines for improving population health and reducing unequal health distribution [12].

The Belgian Health Interview Survey (BHIS) collected self-reported data on chronic diseases and HRQoL in a representative sample of the general Belgian population. Linking HRQoL data with disease prevalence data allows quantifying QALY losses at population level, which is of major interest to many researchers, specifically health economists, clinicians, and policy makers. This study aims to estimate the burden of chronic diseases in terms of QALY losses and to model its social distribution for the general population.

## Methods

### Belgian Health Interview Survey

Data from the BHIS 2013 and 2018 were used. The BHIS is a cross-sectional household survey conducted periodically in Belgium since 1997. The survey provides representative results at the level of the Belgian population. For each survey, approximately 10,000 participants are selected through multistage stratified sampling. In 2013 and 2018, 10,829 and 11,611 individuals were interviewed with a response rate of 57.1% and 57.5% at household level, respectively. Sociodemographic and clinical data were collected through face-to-face interviews, data on HRQoL were assessed via a self-administered written questionnaire in four languages (Dutch, French, German, and English). Details on methodology of the BHIS are described elsewhere [13]. The BHIS covers the entire population, however, this study only considered individuals aged ≥ 15 years.

### Measures

#### Sociodemographic information

The following sociodemographic data were used: age (15 to 101 years), gender (male, female), civil status (single, married or legally cohabiting, widow(er), divorced), region (Brussels, Flanders, Wallonia), and educational attainment (no diploma, lower education, lower secondary education, higher secondary education, post-secondary not-higher education, higher education (academic bachelor or master), doctoral degree). The latter was used as a proxy for SES to assess inequalities in health status. SES was based on the highest level of education achieved in the household and was classified, according to the International Standard Classification of Education [14], into three categories: low (lower secondary education or less; ISCED 0-2), intermediate (higher secondary education; ISCED 3-4), and high (higher education; ISCED 5-6).

#### Chronic diseases

Data on chronic diseases was based on the following question: ‘Have you had one of the following disease or condition in the past 12 months?’. Participants had to indicate on a list of 38 chronic diseases whether they had suffered from a certain disease with the responses ‘yes’ or ‘no’. In addition to chronic diseases, the list also included chronic conditions (i.e. health issues that exceed the scope of the traditional disease model as they do not cause symptoms but may have an impact on clinical care [15]), consequences of chronic diseases, and acute diseases with chronic consequences. The following diseases were included: asthma, chronic bronchitis/COPD/emphysema, myocardial infarction, coronary heart disease, serious heart disease, hypertension, high cholesterol level in blood, stroke, narrowing of blood vessels in belly or legs, rheumatoid arthritis, osteoarthritis, low back disorder, neck disorder, osteoporosis, hip fracture, allergy, cancer, severe headache (e.g., migraine), thyroid problems, diabetes, diabetic retinopathy, glaucoma, cataract, macular degeneration, Parkinson's disease, epilepsy, serious gloom or depression, chronic fatigue, stomach ulcer, cirrhosis of the liver/liver dysfunction, disorder of the larger or the small bowel, stones in the kidney, serious disease of the kidney, chronic cystitis, gallstones or inflammation of the gallbladder, serious or chronic skin disease, urinary incontinence, and prostate problems. These 38 chronic conditions were mapped into 23 chronic diseases or disease groups because many conditions are affecting the same body system. The mapping was based on the International Statistical Classification of Diseases and Related Health Problems 10^th^ Revision (ICD-10) and a multimorbidity questionnaire (MM-21) (Appendix 1) [16, 17].

#### EQ-5D-5L

The EQ-5D-5L was used to assess participants’ HRQoL. The EQ-5D-5L consists of a descriptive system including five health-related dimensions: mobility, self-care, usual activities, pain/discomfort, and anxiety/depression. Each dimension defines five levels (5L) of perceived problems (no problems, slight problems, moderate problems, severe problems, and extreme problems/unable to), from which a single index value or utility score can be calculated anchored by 0 (death) and 1 (perfect health). Negative values can also occur for health states perceived worse than death. Converting the health states into a single EQ-5D index value requires a country-specific algorithm based on population-level preferences for different health states. Recently, an EQ-5D-5L value set has been developed based on health states preferences from the general population of Belgium [18]. Possible index values range between -0.532 (worst health state) and 1 (most optimal health state). The EQ-5D also includes a visual analogue scale (VAS) which measures general health perception on a vertical scale from 0 (worst imaginable health) to 100 (best imaginable health). The EQ-VAS was not used in this study because it was not included in the BHIS 2018.

### Statistical analysis

Statistical analyses were conducted using the IBM SPSS statistical software (version 27.0). For all analyses, the design effects of the survey (i.e. survey weights, clustering at household level, and stratification by province) were applied to deal with the complex design of the BHIS [19]. As such, nationally representative population-based results were generated.

To assess the total burden of chronic diseases at population level, the annual QALY loss per 100,000 individuals was calculated for each condition. The following formula was used:

$$Annual\ QALY\ loss= disease\ prevalence\times HRQoL\ disutility\ score\times \mathrm{100,000}\times 1\ year$$

Disease prevalence estimates were based on the population aged 15 years and older (n=9467 in 2013, n=10,380 in 2018), thus not only from participants who had EQ-5D data. HRQoL disutilities can be regarded as the difference in utility score between a reference population and the chronically ill. Data on the reference population was based on the EQ-5D-5L Belgian population norms [20], i.e. HRQoL data for the average person in the general population in a similar age and/or gender and/or region group. Negative disutilites were equated to zero. The following formula was used:

$$HRQoL\ disutility= HRQoL\ population\ reference\ value- individual\ HRQoL\ value$$

To assess socioeconomic inequalities in QALYs across the whole population, a composite measure was calculated, i.e. the Population Attributable Fractions (PAF) [21]. The PAF is an epidemiological measure to assess the public health impact of exposures in populations. It refers to the fraction of all cases with a particular outcome in a population that is associated with a risk factor. In this study, the PAF indicates which fraction of the QALY loss is associated with the risk factor of low SES. The PAF was calculated as:

$$PAF=\frac{Annual\ QALY\ los{s}_{total\ disease\ population}- Annual\ QALY\ los{s}_{highest\ SES\ group}}{Annual\ QALY\ los{s}_{total\ disease\ population}}$$

## Results

The EQ-5D-5L was completed by 77% of the eligible participants (*n*=6190, mean age 48.4 years, 52% women) in 2013 and by 85% of the eligible participants (*n*=7509, mean age 48.6 years, 52% women) in 2018. Sample characteristics are outlined in Table 1.

**Table 1:** Characteristics of the study participants in 2013 (*N* = 6190) and 2018 (*N* = 7509), survey-weighted

	2013	2018	***P***-value
**Age, mean (SD)**	48.5 (18.47)	48.6 (18.88)	
15-24 years	11.0%	11.7%	<0.001
25-44 years	32.5%	31.6%	
45-64 years	35.0%	35.1%	
≥ 65 years	21.5%	21.6%	
**Sex**			
Female	52.3%	51.6%	<0.001
Male	47.7%	48.4%	
**Socioeconomic status**			
Low	21.8%	16.8%	<0.001
Intermediate	33.7%	32.4%	
High	44.4%	50.8%	
**Civil status**			
Single	26.5%	29.3%	<0.001
Married or legally cohabiting	55.8%	54.3%	
Widow(er)	7.8%	6.7%	
Divorced	10.0%	9.7%	
**Region**			
Flanders	61.7%	58.6%	<0.001
Brussels	7.8%	9.0%	
Wallonia	30.5%	32.4%	

### QALY losses and chronic diseases

**Fig. 1:**
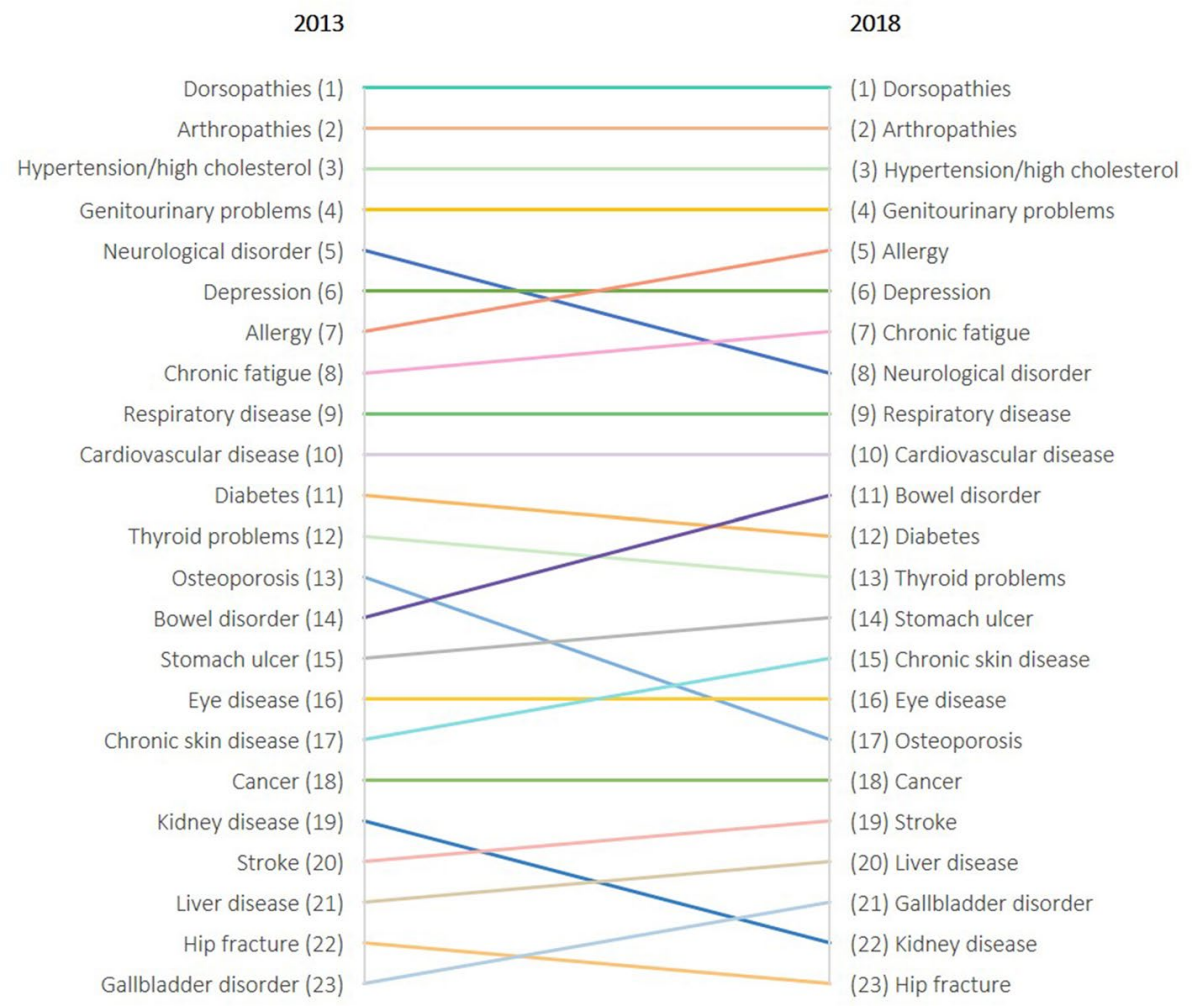
Annual quality-adjusted life-year (QALY) loss per 100,000 individuals by rank 2013 and 2018

Figure 1 presents the ranking of causes of total QALY loss for 2013 and 2018. For both years, the largest QALY loss was observed in dorsopathies, followed by arthropathies, hypertension/high cholesterol, and genitourinary problems. Conversely, the smallest QALY loss was observed in diseases such as gallbladder disorder, hip fracture, liver disease, kidney disease, and stroke. A different pattern was observed while looking at the individual loss in HRQoL, i.e. disutility (Appendix 2). Disutilities were larger for lower rank diseases (e.g. stroke: -0.196 in 2013, -0.324 in 2018; liver disease: -0.277 in 2013, -0.195 in 2018; hip fracture: -0.275 in 2013, -0.185 in 2018), while disutilities were smaller for higher rank diseases (e.g. hypertension/high cholesterol: -0.127 in 2013, -0.130 in 2018; allergy: -0.122 in 2013, -0.135 in 2018). Outliers were reported for depression (-0.285 in 2013, -0.313 in 2018) and chronic fatigue (-0.303 in 2013, -0.278 in 2018), both having the greatest disutilities. The fact that some diseases have a high individual burden but a low total QALY burden at population level is due to a low prevalence. Indeed, QALY losses are not only influenced by the individual HRQoL loss due to disease but also by the disease prevalence. Top-ranked diseases also had the highest prevalence, for example, dorsopathies (24.9% in 2013, 30.1% in 2018) and hypertension/high cholesterol (26.1% in 2013, and 27.7% in 2018).

### QALY losses and gender/age

**Fig. 2:**
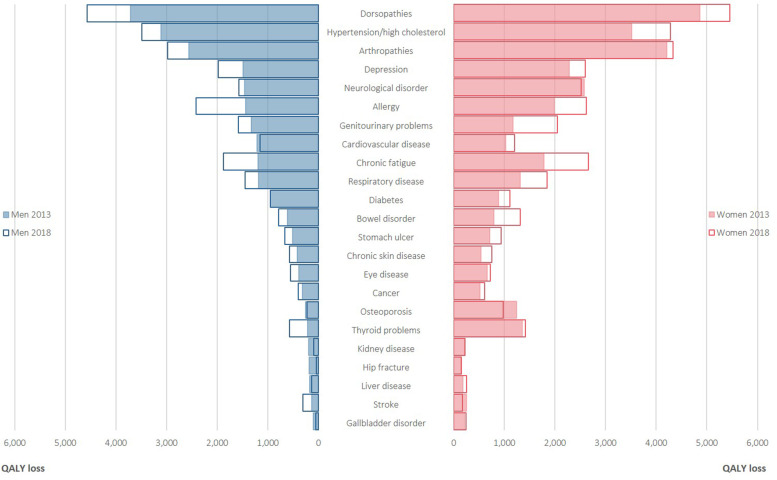
Annual quality-adjusted life-year (QALY) loss per 100,000 individuals associated with 23 chronic diseases, by gender, for the Belgian population aged 15 years and older, 2013 and 2018

**Fig. 3:**
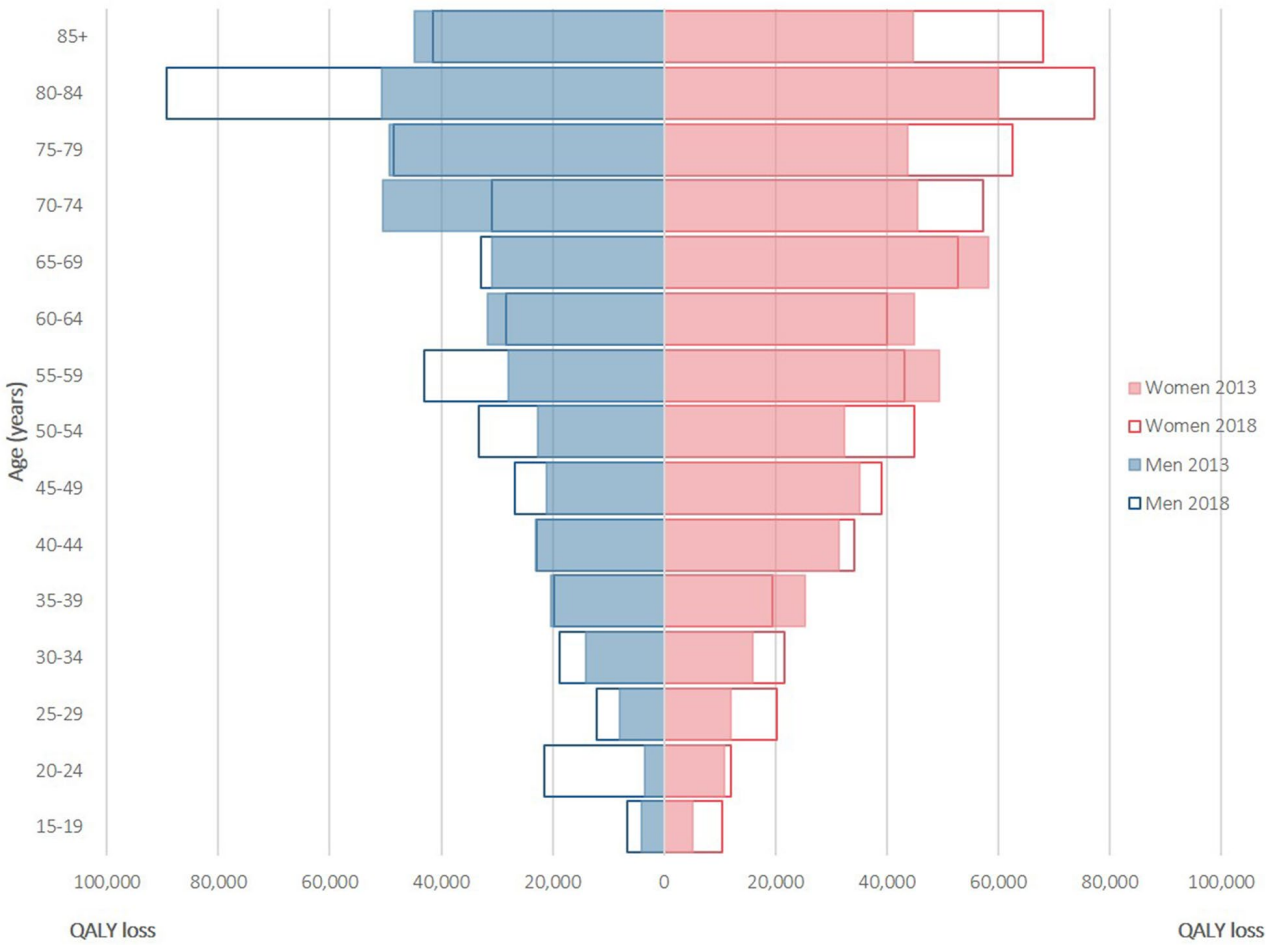
Annual quality-adjusted life-year (QALY) loss per 100,000 individuals for all chronic diseases by gender and age, 2013 and 2018

The annual QALY loss according to gender is depicted in Fig. 2. Overall, QALY losses were larger in women than in men. In women, the largest QALY loss was for dorsopathies (4859 QALYs in 2013, 5458 QALYs in 2018) in both years, followed by arthropathies (4213 QALYs in 2013, 4332 QALYs in 2018) and hypertension/high cholesterol (3521 QALYs in 2013, 4286 QALYs in 2018). In men, the largest QALY loss was for dorsopathies (3711 QALYs in 2013, 4574 QALYs in 2018) in both years, followed by hypertension/high cholesterol (3107 QALYs in 2013, 3490 QALYs in 2018) and arthropathies (2564 QALYs in 2013, 2983 QALYs in 2018). The annual QALY loss according to age is depicted in Fig. 3. Overall, QALY losses were larger in older individuals than in their younger counterparts. In most cases, both prevalence and disutilities increased as the age of the participants increased.

### QALY losses and socioeconomic status

**Fig. 4:**
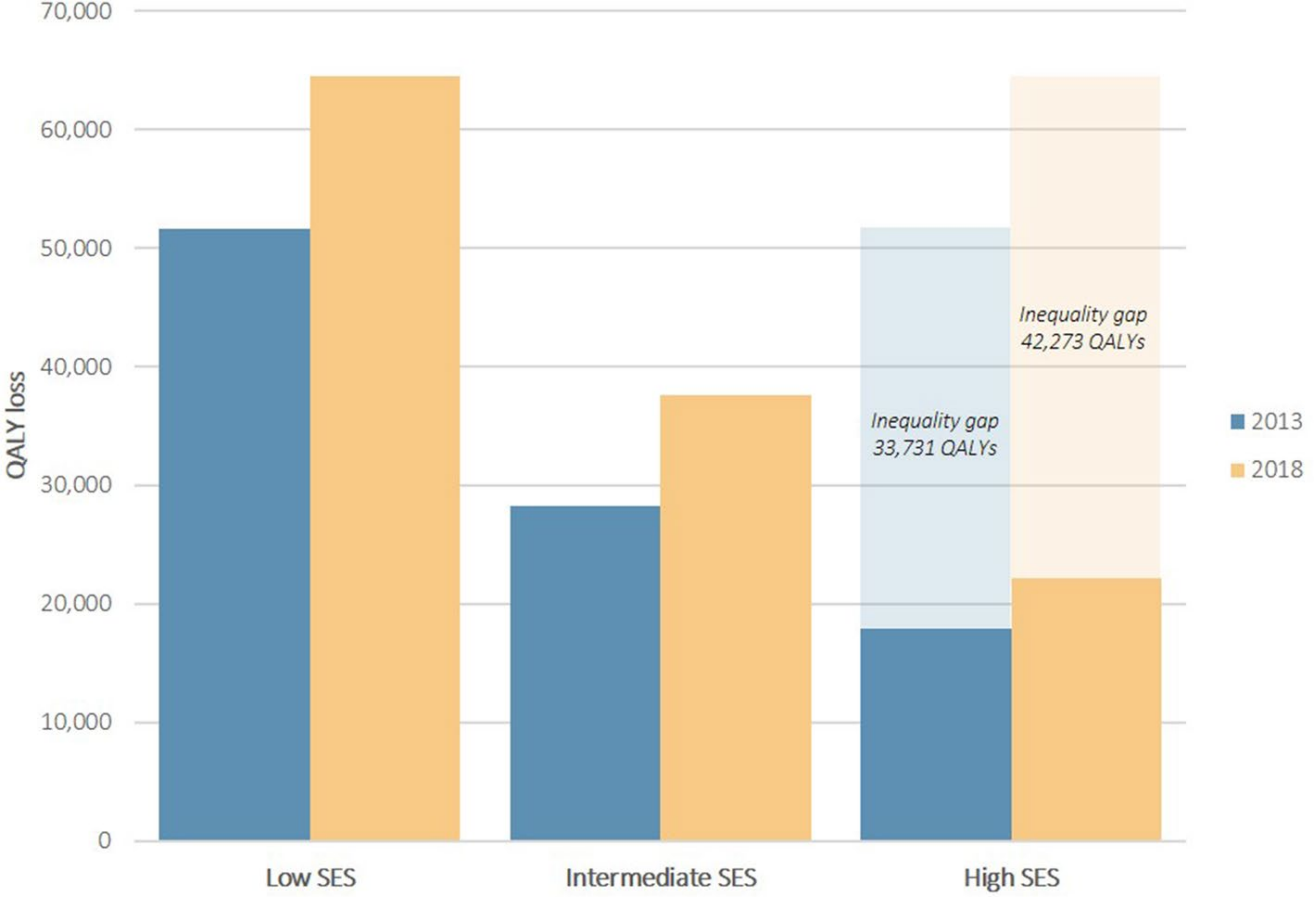
Annual quality-adjusted life-year (QALY) loss per 100,000 individuals for all chronic diseases by socioeconomic status (SES)

The annual QALY loss was also computed by socioeconomic position (Table 2). Individuals with high SES had consistently lower QALY loss when facing a chronic disease compared to individuals with low SES. The difference between high SES and intermediate SES was less pronounced. The level of inequalities can be better understood by the composite measure of PAF. For example, in 2018, a PAF of 74% was found in stroke which means that 74% of the QALY loss could have been avoided if the total population had high SES. In both years, a higher PAF was found in individuals with hip fracture and stroke. In 2013, a negative PAF was reported in allergy (-7%) which means that not having a high SES would reduce the QALY loss by 7%. Figure 4 depicts the annual QALY loss for all chronic diseases by socioeconomic status (SES), with low SES having the largest total QALY loss. In 2013, the inequality gap (low SES versus high SES) amounts to 33,731 QALYs. In 2018, the inequality gap further expanded to 42,273 QALYs.

**Table 2:**
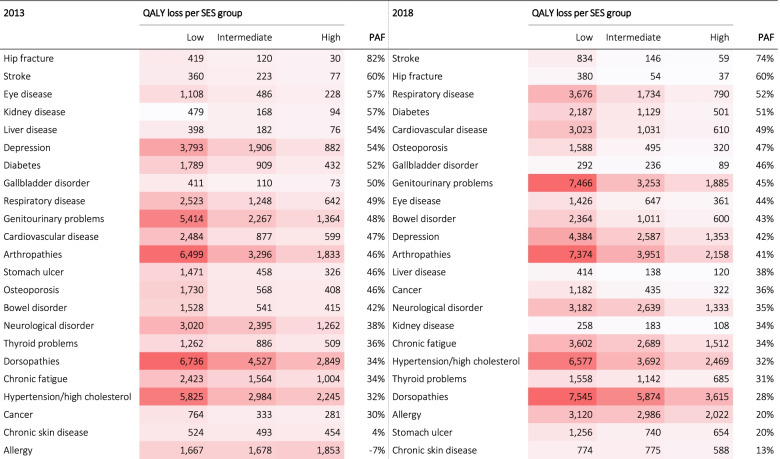
Annual quality-adjusted life-year (QALY) loss per 100,000 individuals by socioeconomic status (SES) and Population Attributable Fractions (PAF), 2013 and 2018

## Discussion

This study examined the overall burden for 23 chronic diseases, chronic conditions, and diseases with chronic consequences in terms of QALY losses, a metric which is widely used in health economic evaluations, based on representative data captured in the BHIS. Moreover, the results of this study provide novel insights into socioeconomic inequalities in QALYs, which is useful to support policy trade-offs between improving population health and reducing unequal health distribution. In general, this study provides evidence for the need for health policies targeting chronic diseases in the most vulnerable populations, i.e. women, older persons, and low SES populations.

We stratified our findings by age, sex, SES, and time point. In 2013 and 2018, the largest QALY loss was due to dorsopathies, arthropathies, genitourinary problems, and hypertension/high cholesterol. Earlier research indicated that musculoskeletal disorders and hypertension were associated with the largest loss of QALYs in the population [1, 22]. Comparable with previous research, these results are mainly attributed to their high prevalence among the Belgian population [1, 23]. This study also recognizes the substantial impact of psychological disorders (i.e. depression as the 5^th^ cause of QALY loss in 2018) due to greatly impaired HRQoL. Moreover, the QALY losses for different age intervals revealed that older age groups are most affected by chronic diseases as expected [24]. Furthermore, women had a larger QALY loss than men which can be mainly attributed to higher disutility values in women [25, 26]. This study also showed that the burden is higher in 2018 compared to 2013 because of increases in disease prevalence as a result of population ageing. Chronic diseases are paramount in an ageing society and susceptibility to chronic diseases increases with age [27]. More importantly, a large inequality gap in QALY losses was found between the least and the most deprived population groups, which is consistent with previous research [12, 28]. Indeed, SES is the main determinant of chronic disease distribution in populations [29]. The largest inequality gap was seen in arthropathies and hypertension/high cholesterol, mainly due to higher prevalence rates in low SES groups. Low SES is indeed found to be associated with the risk of developing arthritis and hypertension due to higher smoking rates, body mass index (BMI), and lack of exercise compared to high SES groups [30–32]. There is also strong evidence that SES is associated with worse HRQoL outcomes [25, 33, 34]. Hence, it is expected that the inequality gap in QALY loss due to chronic diseases will continue to grow. It is important to mention that the increase in PAF can be partly explained by population aging.

Several limitations related to the BHIS should be acknowledged. First, information on chronic morbidity was based on self-reports measured by a single and global question. The accuracy of self-reports depends on the participants’ knowledge and understanding of the relevant information, ability to recall it, and willingness to report it [35]. This is challenging because participants are often confused to distinguish between symptoms and the actual disease, and because some diseases are very subjective (e.g. chronic fatigue). In addition, people may indicate to have several diseases (e.g. depression and chronic fatigue) because both diseases have homogeneous symptoms and common etiology. Although self-reported chronic morbidity may underestimate the prevalence of medical conditions (thus underestimating QALY losses), it is found to be a reasonably reliable instrument to measure ill health [36]. Another limitation is the incomplete list of chronic diseases included in the BHIS, implying potential missing of other important chronic conditions. Besides, few mental or psychiatric conditions were included. Another limitation is potential selection bias, which may result from educational differences in survey participation and in the willingness and ability to answer the self-administered questionnaire. Accordingly, lower participation rates were found in lower educated households, especially when they have a poor health status and a risky health behaviour compared with higher educated households [37, 38]. Consequently, health inequalities may be underestimated in the present study. Moreover, the definition of SES is debatable as it only includes educational attainment. Indeed, income or employment status are also important indicators of SES. Nevertheless, these indicators were not used because information on these variables was less frequently available [39]. However, educational attainment is found to be a relatively stable measure of SES and is usually of good quality [40, 41].

Some methodological considerations should be mentioned. First, we estimated the disease prevalence in all respondents and not only in those who completed the EQ-5D. As such, the estimated prevalence corresponds better with the actual prevalence in the general population. A second methodological issue is related to the calculation of disutilities. In general, when the HRQoL score of a respondent is higher than the general population norm, the difference results in negative values (i.e. gain in HRQoL), which is methodologically irrelevant. As such, we replaced negative values by zero. Third, the possible effects of comorbidity were not taken into account when calculating QALY loss, examining the impact of combinations of conditions would provide a more dynamic and comprehensive overview, especially in older age categories. Fourth, cross-country comparisons of QALY losses are difficult due to differences in EQ-5D value sets resulting from sociocultural differences [42]. It is therefore recommended to compare and interpret QALY outcomes, and cost-utility outcomes in general, from different countries with caution [43]. Fifth, we did not conduct statistical testing given the descriptive nature of this study. However, additional analysis may be considered in future research.

These limitations notwithstanding, this study provides representative results at the level of the Belgian population. In addition, we used the health status of the general population as comparator when estimating HRQoL loss. Using ‘perfect health’ as comparator would have resulted in an overestimation of QALY losses. The current economic standard is to elicit and compare HRQoL estimated from the general public because economic evaluations are meant to guide social policies [44].

## Conclusions

This study estimated the burden of chronic diseases in terms of QALY losses and modeled its social distribution for the general population. Given that chronic diseases will rise in the next decades, addressing its burden is necessary, particularly among the most vulnerable (i.e. older persons, women, low SES). Interventions in these target groups are preferentially required in order to reduce the burden of chronic diseases.

## Supplementary Information

**Additional file 1.** Appendix 1.

**Additional file 2.** Appendix 2.

## Data Availability

Access to micro data of the BHIS can be requested via https://his.wiv-isp.be.

## Abbreviations

BHIS: Belgian Health Interview Survey
HRQoL: Health-related quality of life
ICD-10: International Statistical Classification of Diseases and Related Health Problems 10th Revision (ICD-10)
ISCED: International Standard Classification of Education
PAF: Population attributable fractions
QALY: Quality-adjusted life year
SES: Socioeconomic status
VAS: Visual analogue scale
