# Supplementary material for: QALY losses for chronic diseases and its social distribution in the general population: results from the Belgian Health Interview Survey
Source: BMC Public Health. 2022 Jul 7;22:1304. doi: 10.1186/s12889-022-13675-y (PMC9264606; doi:10.1186/s12889-022-13675-y)
Supplement: Supplementary file 1 — Additional file 1. Appendix 1. [file 12889_2022_13675_MOESM1_ESM.docx]

| **Appendix 1.** Mapping of the chronic diseases according to the MM-21 and ICD-10. | | |
| --- | --- | --- |
| **BHIS 2018** (38 chronic diseases) |  | **MM-21/ICD-10** (23 chronic diseases) |
|  | | |
| Allergy |  | Allergy (T78.4, J30.1) |
|  |  |  |
|  |  |  |
| Hip fracture |  | Hip fracture (S70-S79) |
|  |  |  |
|  | | |
| Cancer |  | Cancer (C00-D49) |
|  |  |  |
|  | | |
| Chronic fatigue |  | Chronic fatigue (R53) |
|  |  |  |
|  | | |
| Chronic skin disease |  | Chronic skin disease (L80-L9) |
|  |  |  |
|  | | |
| Cirrhosis of the liver/liver dysfunction |  | Cirrhosis of the liver (K70-K77) |
|  |  |  |
|  | | |
| Disorder of the larger or the small bowel |  | Bowel disorder (K50-K52, K57, K58) |
|  |  |  |
|  | | |
| Gallstones of inflammation of the gallbladder |  | Gallstones (K80-K87) |
|  |  |  |
|  | | |
| Osteoporosis |  | Osteoporosis (M81) |
|  |  |  |
|  | | |
| Serious disease of the kidney |  | Kidney disease (N18, N19) |
|  |  |  |
|  | | |
| Serious gloom or depression |  | Depression (F33, F40, F41) |
|  |  |  |
|  | | |
| Stomach ulcer |  | Stomach disorder (K21, K25.7, K29.5) |
|  |  |  |
|  | | |
| Stroke (or consequences) |  | Stroke (G45, I60-I69) |
|  |  |  |
|  | | |
| Thyroid problems |  | Thyroid problems (E00-E07) |
|  |  |  |
|  | | |
| Hypertension  High cholesterol level in blood |  | Hypertension/high cholesterol (I10-I15, E78) |
|  |  |  |
|  |  |  |
| Low back disorder  Neck disorder |  | Dorsopathies (M40-M54, M60-M63, M65-M68, M70-M79) |
|  |  |  |
|  | | |
| Rheumatoid arthritis  Osteoarthritis/arthrosis |  | Arthropathies (M05.9, M13.0, M13.9, M15-M19) |
|  |  |  |
|  | | |
| Asthma  Chronic bronchitis/COPD/emphysema |  | Respiratory disease (J40-J47) |
|  |  |  |
|  | | |
| Diabetes  Diabetic retinopathy |  | Diabetes (E10-E14) |
|  |  |  |
|  | | |
| Epilepsy  Parkinson’s disease  Severe headache (e.g. migraine) |  | Neurological disorder (G40, G20,G43) |
|  |  |  |
|  | | |
| Macular degeneration  Glaucoma  Cataract |  | Eye disease (H35, H40-H42, H25, H26, H28) |
|  |  |  |
|  | | |
| Myocardial infarction  Narrowing of blood vessels in belly or legs  Serious heart disease  Coronary heart disease |  | Cardiovascular disease (I20-I25, I48, I70-I79) |
|  |  |  |
|  | | |
| Urinary incontinence  Stones in the kidney  Chronic cystitis  Prostate problems |  | Genitourinary problems (N03, N11, N18, N20-N23, N25-N29, N30-N39, N40-N51) |
|  |  |  |
