# Supplementary material for: QALY losses for chronic diseases and its social distribution in the general population: results from the Belgian Health Interview Survey
Source: BMC Public Health. 2022 Jul 7;22:1304. doi: 10.1186/s12889-022-13675-y (PMC9264606; doi:10.1186/s12889-022-13675-y)
Supplement: Supplementary file 2 — Additional file 2. Appendix 2. [file 12889_2022_13675_MOESM2_ESM.docx]

**Appendix 2.** Annual quality-adjusted life-year (QALY) loss per 100,000 individuals associated with 23 chronic diseases, 2013 and 2018

| **2013** | **QALY loss** | **Disutility** | **Prevalence** | **2018** | **QALY loss** | **Disutility** | **Prevalence** |
| --- | --- | --- | --- | --- | --- | --- | --- |
| Dorsopathies | 4,305 | -0.173 | 0.249 | Dorsopathies | 5,033 | -0.167 | 0.301 |
| Arthropathies | 3,414 | -0.175 | 0.195 | Arthropathies | 3,674 | -0.173 | 0.212 |
| Hypertension/high cholesterol | 3,320 | -0.127 | 0.261 | Hypertension/high cholesterol | 3,612 | -0.130 | 0.277 |
| Genitourinary problems | 2,630 | -0.189 | 0.139 | Genitourinary problems | 3,418 | -0.210 | 0.163 |
| Neurological disorder | 2,035 | -0.196 | 0.104 | Allergy | 2,528 | -0.135 | 0.187 |
| Depression | 1,912 | -0.285 | 0.067 | Depression | 2,316 | -0.313 | 0.074 |
| Allergy | 1,725 | -0.122 | 0.142 | Chronic fatigue | 2,278 | -0.278 | 0.082 |
| Chronic fatigue | 1,513 | -0.303 | 0.050 | Neurological disorder | 2,058 | -0.181 | 0.114 |
| Respiratory disease | 1,262 | -0.178 | 0.071 | Respiratory disease | 1,643 | -0.191 | 0.086 |
| Cardiovascular disease | 1,131 | -0.199 | 0.057 | Cardiovascular disease | 1,186 | -0.198 | 0.060 |
| Diabetes | 906 | -0.156 | 0.058 | Bowel disorder | 1,044 | -0.227 | 0.046 |
| Thyroid problems | 798 | -0.138 | 0.058 | Diabetes | 1,028 | -0.166 | 0.062 |
| Osteoporosis | 759 | -0.185 | 0.041 | Thyroid problems | 992 | -0.142 | 0.070 |
| Bowel disorder | 713 | -0.255 | 0.028 | Stomach ulcer | 815 | -0.227 | 0.036 |
| Stomach ulcer | 607 | -0.225 | 0.027 | Chronic skin disease | 674 | -0.173 | 0.039 |
| Eye disease | 531 | -0.136 | 0.039 | Eye disease | 649 | -0.147 | 0.044 |
| Chronic skin disease | 472 | -0.169 | 0.028 | Osteoporosis | 606 | -0.184 | 0.033 |
| Cancer | 403 | -0.212 | 0.019 | Cancer | 505 | -0.211 | 0.024 |
| Kidney disease | 219 | -0.219 | 0.010 | Stroke | 227 | -0.324 | 0.007 |
| Stroke | 196 | -0.196 | 0.010 | Liver disease | 195 | -0.195 | 0.010 |
| Liver disease | 166 | -0.277 | 0.006 | Gallbladder disorder | 165 | -0.150 | 0.011 |
| Hip fracture | 165 | -0.275 | 0.006 | Kidney disease | 164 | -0.164 | 0.010 |
| Gallbladder disorder | 148 | -0.211 | 0.007 | Hip fracture | 93 | -0.185 | 0.005 |
